# Poly[μ-2,3-dihydroxy­propan-1-olato-sodium]

**DOI:** 10.1107/S1600536810016077

**Published:** 2010-05-12

**Authors:** Gabriele Schatte, Jianheng Shen, Martin Reaney, Ramaswami Sammynaiken

**Affiliations:** a Saskatchewan Structural Sciences Centre, University of Saskatchewan, Saskatoon, Saskatchewan, Canada S7N 5C9; b Department of Food and Bioresources, University of Saskatchewan, Saskatoon, Saskatchewan, Canada S7N 5A8

## Abstract

The Na^+^ cation in the title compound, [Na(C_3_H_7_O_3_)]_*n*_ or Na[H_2_gl], is coordinated by five O atoms leading to a distorted trigonal-bipyramidal geometry. The negatively charged O atom of the glycerolate anion is in an equatorial position, and the O atom of the hydroxo group, attached to the secondary C atom, occupies an axial position completing a five-membered non-planar chelate ring; this defines the asymmetric unit. The Na^+^ cation is coordinated by three other symmetry-related monodentate H_2_gl^−^ ligands, so that each H_2_gl^−^ ligand is bonded to four Na^+^ ions. The H_2_gl^−^ ligands are connected *via* strong O—H⋯O hydrogen bonds and these, together with the Na⋯O inter­connections, are responsible for the formation of polymeric sheets which propagate in the directions of the *b* and *c* axes.

## Related literature

For syntheses of mono sodium glyceroxide, see: Letts (1872 ▶); Fairbourne & Toms (1921 ▶); Gross & Jacobs (1926 ▶). For the syntheses and characterization of sodium alkoxides and aryl­oxides, see: Davies *et al.* (1982 ▶); Brooker *et al.* (1991 ▶); Hogerheide *et al.* (1996 ▶). For related crystal structures of transition metal mono glyceroxides, see: Rath *et al.* (1998 ▶). 
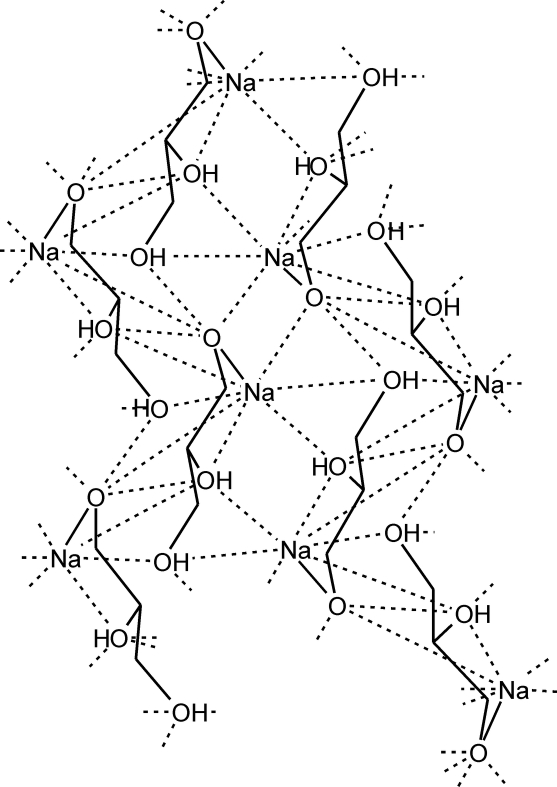


## Experimental

### 

#### Crystal data

[Na(C_3_H_7_O_3_)]
*M* *_r_* = 114.08
Monoclinic, 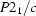

*a* = 8.1117 (4) Å
*b* = 6.1559 (3) Å
*c* = 9.4882 (5) Å
β = 100.113 (3)°
*V* = 466.43 (4) Å^3^
*Z* = 4
Mo *K*α radiation
μ = 0.22 mm^−1^
*T* = 183 K
0.25 × 0.25 × 0.13 mm

#### Data collection

Bruker–Nonius KappaCCD four-circle diffractometer
Absorption correction: multi-scan (*SCALEPACK*; Otwinowski & Minor, 1997 ▶) *T*_min_ = 0.948, *T*_max_ = 0.972
1963 measured reflections
1058 independent reflections
953 reflections with *I* > 2σ(*I*)
*R*_int_ = 0.016

#### Refinement

*R*[*F* ^2^ > 2σ(*F* ^2^)] = 0.026
*wR*(*F* ^2^) = 0.068
*S* = 1.07
1058 reflections
72 parameters
H atoms treated by a mixture of independent and constrained refinement
Δρ_max_ = 0.34 e Å^−3^
Δρ_min_ = −0.22 e Å^−3^

### 

Data collection: *COLLECT* (Nonius, 1998 ▶); cell refinement: *DENZO/*SCALEPACK** (Otwinowski & Minor, 1997 ▶); data reduction: *DENZO/*SCALEPACK**; program(s) used to solve structure: *SIR97* (Altomare *et al.*, 1999 ▶); program(s) used to refine structure: *SHELXL97* (Sheldrick, 2008 ▶); molecular graphics: *CAMERON* (Watkin *et al.*, 1993 ▶) and *ORTEP* (in *SHELXTL-NT*; Sheldrick, 2008 ▶); software used to prepare material for publication: *publCIF* (Westrip, 2010 ▶).

## Supplementary Material

Crystal structure: contains datablocks global, I. DOI: 10.1107/S1600536810016077/tk2652sup1.cif

Structure factors: contains datablocks I. DOI: 10.1107/S1600536810016077/tk2652Isup2.hkl

Additional supplementary materials:  crystallographic information; 3D view; checkCIF report

## Figures and Tables

**Table 1:** Selected bond lengths (Å)

O1—Na1	2.4243 (10)
O2—Na1	2.4237 (9)
Na1—O1^i^	2.3163 (9)
Na1—O3^ii^	2.3462 (10)
Na1—O2^iii^	2.3551 (9)
Na1—O2^ii^	3.3549 (10)
Na1—O3^iii^	3.5265 (10)
Na1—O1^iv^	3.8258 (10)

Symmetry codes: (i) 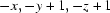; (ii) 
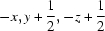
; (iii) 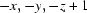; (iv) 
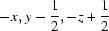
.

**Table 2:** Hydrogen-bond geometry (Å, °)

*D*—H⋯*A*	*D*—H	H⋯*A*	*D*⋯*A*	*D*—H⋯*A*
O2—H2⋯O1^iv^	0.865 (18)	1.723 (18)	2.5837 (12)	173.0 (18)
O3—H3⋯O1^v^	0.857 (18)	1.804 (18)	2.6575 (12)	173.7 (19)

Symmetry codes: (iv) 
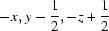
; (v) 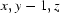.

**Fig. 1.**
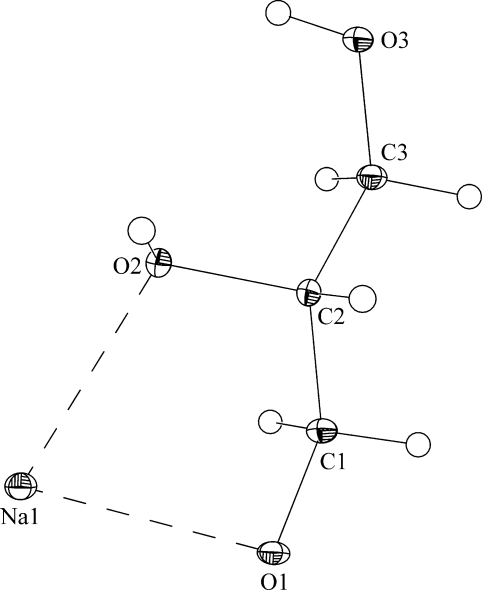
Molecular structure of the asymmetric unit in (I) showing the labelling scheme. Non-hydrogen atoms are represented by displacement ellipsoids at the 30% propability level.

**Fig. 2.**
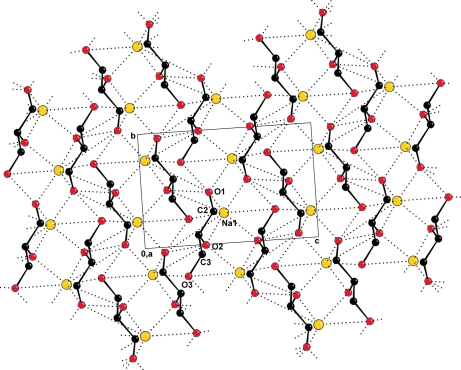
Partial packing diagram for (I) showing the intra- and inter-molecular Na···O and intermolecular O(H)···O contacts (dashed lines) leading to a polymeric sheet-like structure. Hydrogen atoms have been omitted for clarity.

## supplementary crystallographic information

### Comment

We have shown that alkali metal glyceroxides can be used as efficient catalysts in *trans*-esterification reactions to produce biodiesel. Earlier syntheses of the mono sodium glyceroxide, Na[OCH_2_CH(OH)CH_2_(OH)] (referred to as Na[H_2_gl]), involved the reaction of excess sodium dissolved in ethanol with glycerol (Letts, 1872; Fairbourne & Toms, 1921). A more elegant and less expensive method for the the preparation of the title compound, (I), involved heating and stirring together equimolar quantities of powdered sodium hydroxide and glycerol (Gross & Jacobs, 1926).

Crystal structures of putative alkali metal glycerolates, M[H_2_gl], have not been reported to our knowledge. The crystal structure of oxo(propane-1,3-diol-2-olato)(salicylaldehyde hydroxophenylmethylenehydrazonato-N,*O*,*O*')vanadium(V) has been until now the only reported structure containing coordinated H_2_gl^-^ ions (Rath *et al.*, 1998). The crystal structure of (I) was determined as part of our research on catalysts which can be used in the production of biodiesel. The results of our crystal structure determination confirmed the earlier proposed structure based on derivative chemistry (Fairbourne & Toms, 1921).

The H_2_gl^-^ anion behaves as a multifunctional ligand in the structure of (I), Fig. 1. In the first mode, the H_2_gl^-^ ligand is coordinating to the sodium atom by one oxo- (O1) and one hydroxo (O2) group forming a non-planar 5-membered ring. Symmetry related H_2_gl^-^ ligands form essentially monodentate attachments. Pseudo-five-membered chelate rings are formed if rather longer Na···O interactions are taken into account [Na···O distances ranging from 3.35 to 3.83 Å (sum of the van der Waals radii, 3.8 Å)]; Table 1 and Fig. 2.

The observed intra- and inter-molecular Na···O bond distances are elongated in comparison to the related bond distances reported for sodium phenolate complexes (Hogerheide *et al.*, 1996; Brooker *et al.*, 1991) and sodium *tert*-butoxide (Davies *et al.*, 1982). The oxygen atoms O1 and O2 act as bridging atoms between sodium atoms forming a planar O···Na···O···Na ring with alternation between O1 in one ring and O2 in the following ring. Each H_2_gl^-^ ligand is bonded to four Na ions. The H_2_gl^-^ ligands are connected *via* two strong intermolecular O—H···O hydrogen bond interactions (Table 2 and Fig. 2). Both the Na···O and O—H···O interconnections are responsible for the formation of polymeric sheets which extends indefinitely in the directions of the *b* and *c* axes (Fig. 2). Finally, it is noted that in (I), the hydroxo group attached to primary carbon atom of the glycerol is deprotonated. This is in contrast to the reported structure for the vanadium-H_2_gl complex, where the hydroxo group attached to secondary carbon atom is deprotonated (Rath *et al.*, 1998).

### Experimental

A sodium hydroxide solution (240 g, 50%) was freshly prepared by dissolving sodium hydroxide pellets (120 g, 3 mol) in water (120 g). Glycerol (92 g, 1 mol) was slowly added into the hot sodium hydroxide solution under agitation. The mixture was allowed to stand and to cool down to room temperature. Colourless crystals of mono sodium glyceroxide started to form. The crystals are only stable in a very basic solution at ambient temperatures. A suitable single crystal was quickly coated with oil, collected onto the nylon fiber of a mounted CryoLoop^TM^ and quickly transferred to the cold stream of the X-ray diffractometer. The data collection was performed at -90°C instead of -100°C to prevent cracking of the crystals at the lower temperature.

### Refinement

The C-bound H atoms were geometrically placed (C–H = 0.98–1.00 Å) and refined as riding with *U_iso_*(H) = 1.2*U_eq_*(parent atom). The hydrogen atoms of the hydroxo groups were located in the difference Fourier map and were allowed to refine freely.

### Crystal data

**Table:**

[Na(C_3_H_7_O_3_)]	*F*(000) = 240
*M**_r_* = 114.08	*D*_x_ = 1.624 Mg m^−^^3^
Monoclinic, *P*2_1_/*c*	Mo *K*α radiation, λ = 0.71073 Å
Hall symbol: -P 2ybc	Cell parameters from 1052 reflections
*a* = 8.1117 (4) Å	θ = 1.0–27.5°
*b* = 6.1559 (3) Å	µ = 0.22 mm^−^^1^
*c* = 9.4882 (5) Å	*T* = 183 K
β = 100.113 (3)°	Plate, colourless
*V* = 466.43 (4) Å^3^	0.25 × 0.25 × 0.13 mm
*Z* = 4	

### Data collection

**Table:**

Bruker–Nonius KappaCCD four-circle diffractometer	1058 independent reflections
Radiation source: fine-focus sealed tube	953 reflections with *I* > 2σ(*I*)
horizonally mounted graphite crystal	*R*_int_ = 0.016
Detector resolution: 9 pixels mm^-1^	θ_max_ = 27.5°, θ_min_ = 4.0°
ω scans with κ offsets	*h* = −10→10
Absorption correction: multi-scan (*SCALEPACK*; Otwinowski & Minor, 1997)	*k* = −7→7
*T*_min_ = 0.948, *T*_max_ = 0.972	*l* = −12→12
1963 measured reflections	

### Refinement

**Table:**

Refinement on *F*^2^	Primary atom site location: structure-invariant direct methods
Least-squares matrix: full	Secondary atom site location: difference Fourier map
*R*[*F*^2^ > 2σ(*F*^2^)] = 0.026	Hydrogen site location: inferred from neighbouring sites
*wR*(*F*^2^) = 0.068	H atoms treated by a mixture of independent and constrained refinement
*S* = 1.07	*w* = 1/[σ^2^(*F*_o_^2^) + (0.0234*P*)^2^ + 0.217*P*] where *P* = (*F*_o_^2^ + 2*F*_c_^2^)/3
1058 reflections	(Δ/σ)_max_ < 0.001
72 parameters	Δρ_max_ = 0.34 e Å^−^^3^
0 restraints	Δρ_min_ = −0.22 e Å^−^^3^
0 constraints	

### Special details

**Table:**

Geometry. All esds (except the esd in the dihedral angle between two l.s. planes) are estimated using the full covariance matrix. The cell esds are taken into account individually in the estimation of esds in distances, angles and torsion angles; correlations between esds in cell parameters are only used when they are defined by crystal symmetry. An approximate (isotropic) treatment of cell esds is used for estimating esds involving l.s. planes.
Refinement. Refinement of *F*^2^ against ALL reflections. The weighted *R*-factor wR and goodness of fit *S* are based on *F*^2^, conventional *R*-factors *R *are based on *F*, with *F* set to zero for negative *F*^2^. The threshold expression of *F*^2^ > σ(*F*^2^) is used only for calculating *R*-factors(gt) etc. and is not relevant to the choice of reflections for refinement. *R*-factors based on *F*^2^ are statistically about twice as large as those based on *F*, and *R*- factors based on ALL data will be even larger.

### Fractional atomic coordinates and isotropic or equivalent isotropic displacement parameters (Å^2^)

**Table:**

	*x*	*y*	*z*	*U*_iso_*/*U*_eq_	
O1	0.14472 (10)	0.45332 (13)	0.38811 (9)	0.01321 (19)	
O2	0.06200 (10)	−0.00441 (13)	0.35060 (9)	0.01191 (19)	
H2	−0.009 (2)	−0.029 (3)	0.273 (2)	0.041 (5)*	
O3	0.29537 (11)	−0.26839 (14)	0.23818 (9)	0.0148 (2)	
H3	0.248 (2)	−0.352 (3)	0.292 (2)	0.039 (5)*	
C1	0.26407 (14)	0.28434 (18)	0.40749 (12)	0.0131 (2)	
H1A	0.2830	0.2379	0.5089	0.016*	
H1B	0.3715	0.3403	0.3864	0.016*	
C2	0.21073 (13)	0.08815 (18)	0.31254 (12)	0.0112 (2)	
H2A	0.1843	0.1381	0.2108	0.013*	
C3	0.34809 (14)	−0.08287 (19)	0.32497 (13)	0.0145 (2)	
H3A	0.4485	−0.0189	0.2951	0.017*	
H3B	0.3792	−0.1287	0.4262	0.017*	
Na1	−0.08654 (6)	0.26587 (7)	0.46569 (5)	0.01393 (15)	

### Atomic displacement parameters (Å^2^)

**Table:**

	*U*^11^	*U*^22^	*U*^33^	*U*^12^	*U*^13^	*U*^23^
O1	0.0163 (4)	0.0087 (4)	0.0148 (4)	0.0023 (3)	0.0031 (3)	−0.0006 (3)
O2	0.0120 (4)	0.0120 (4)	0.0125 (4)	−0.0011 (3)	0.0040 (3)	0.0004 (3)
O3	0.0188 (4)	0.0101 (4)	0.0169 (4)	−0.0001 (3)	0.0066 (3)	−0.0019 (3)
C1	0.0140 (5)	0.0101 (5)	0.0150 (5)	0.0012 (4)	0.0015 (4)	−0.0006 (4)
C2	0.0118 (5)	0.0105 (5)	0.0121 (5)	−0.0004 (4)	0.0043 (4)	0.0008 (4)
C3	0.0134 (5)	0.0109 (5)	0.0195 (6)	0.0004 (4)	0.0035 (4)	−0.0025 (4)
Na1	0.0186 (3)	0.0108 (2)	0.0133 (2)	0.00036 (17)	0.00535 (18)	−0.00017 (17)

### Geometric parameters (Å, °)

**Table:**

O1—C1	1.4108 (13)	C2—C3	1.5224 (15)
O1—Na1	2.4243 (10)	C2—H2A	1.0000
O2—C2	1.4366 (13)	C3—H3A	0.9900
O2—Na1	2.4237 (9)	C3—H3B	0.9900
O2—H2	0.868 (19)	Na1—O1^i^	2.3163 (9)
O3—C3	1.4288 (14)	Na1—O3^ii^	2.3462 (10)
O3—H3	0.86 (2)	Na1—O2^iii^	2.3551 (9)
C1—C2	1.5233 (15)	Na1—O2^ii^	3.3549 (10)
C1—Na1	2.9929 (13)	Na1—O3^iii^	3.5265 (10)
C1—H1A	0.9900	Na1—O1^iv^	3.8258 (10)
C1—H1B	0.9900		
			
C1—O1—Na1^i^	132.80 (7)	O2—C2—H2A	108.5
C1—O1—Na1	99.17 (7)	C3—C2—H2A	108.5
Na1^i^—O1—Na1	85.61 (3)	C1—C2—H2A	108.5
C2—O2—Na1^iii^	119.44 (6)	O3—C3—C2	111.54 (9)
C2—O2—Na1	110.30 (6)	O3—C3—H3A	109.3
Na1^iii^—O2—Na1	96.88 (3)	C2—C3—H3A	109.3
C2—O2—H2	108.5 (13)	O3—C3—H3B	109.3
Na1^iii^—O2—H2	118.0 (13)	C2—C3—H3B	109.3
Na1—O2—H2	101.2 (13)	H3A—C3—H3B	108.0
C3—O3—Na1^iv^	120.20 (7)	O1^i^—Na1—O3^ii^	111.55 (4)
C3—O3—H3	104.9 (13)	O1^i^—Na1—O2^iii^	93.81 (3)
Na1^iv^—O3—H3	102.2 (12)	O3^ii^—Na1—O2^iii^	120.25 (3)
O1—C1—C2	112.98 (9)	O1^i^—Na1—O2	162.14 (4)
O1—C1—Na1	53.10 (5)	O3^ii^—Na1—O2	84.89 (3)
C2—C1—Na1	84.18 (6)	O2^iii^—Na1—O2	83.12 (3)
O1—C1—H1A	109.0	O1^i^—Na1—O1	94.39 (3)
C2—C1—H1A	109.0	O3^ii^—Na1—O1	106.12 (3)
Na1—C1—H1A	78.4	O2^iii^—Na1—O1	125.50 (3)
O1—C1—H1B	109.0	O2—Na1—O1	73.59 (3)
C2—C1—H1B	109.0	O1^i^—Na1—C1	112.47 (3)
Na1—C1—H1B	161.7	O3^ii^—Na1—C1	115.04 (4)
H1A—C1—H1B	107.8	O2^iii^—Na1—C1	101.71 (3)
O2—C2—C3	109.99 (9)	O2—Na1—C1	51.63 (3)
O2—C2—C1	109.25 (9)	O1—Na1—C1	27.73 (3)
C3—C2—C1	111.99 (9)		
			
Na1^i^—O1—C1—C2	155.38 (7)	Na1^iii^—O2—Na1—O1	130.18 (4)
Na1—O1—C1—C2	62.86 (9)	C2—O2—Na1—C1	−14.05 (6)
Na1^i^—O1—C1—Na1	92.51 (8)	Na1^iii^—O2—Na1—C1	110.87 (4)
Na1^iii^—O2—C2—C3	35.83 (11)	C1—O1—Na1—O1^i^	132.68 (7)
Na1—O2—C2—C3	146.65 (7)	Na1^i^—O1—Na1—O1^i^	0.0
Na1^iii^—O2—C2—C1	−87.48 (9)	C1—O1—Na1—O3^ii^	−113.39 (6)
Na1—O2—C2—C1	23.34 (10)	Na1^i^—O1—Na1—O3^ii^	113.93 (4)
O1—C1—C2—O2	−63.28 (12)	C1—O1—Na1—O2^iii^	34.84 (8)
Na1—C1—C2—O2	−17.61 (7)	Na1^i^—O1—Na1—O2^iii^	−97.85 (4)
O1—C1—C2—C3	174.62 (9)	C1—O1—Na1—O2	−33.86 (6)
Na1—C1—C2—C3	−139.72 (8)	Na1^i^—O1—Na1—O2	−166.54 (4)
Na1^iv^—O3—C3—C2	25.87 (12)	Na1^i^—O1—Na1—C1	−132.68 (7)
O2—C2—C3—O3	56.91 (12)	O1—C1—Na1—O1^i^	−52.49 (8)
C1—C2—C3—O3	178.59 (9)	C2—C1—Na1—O1^i^	−177.05 (6)
C2—O2—Na1—O1^i^	−43.92 (14)	O1—C1—Na1—O3^ii^	76.71 (7)
Na1^iii^—O2—Na1—O1^i^	81.00 (12)	C2—C1—Na1—O3^ii^	−47.85 (7)
C2—O2—Na1—O3^ii^	113.73 (7)	O1—C1—Na1—O2^iii^	−151.64 (6)
Na1^iii^—O2—Na1—O3^ii^	−121.34 (4)	C2—C1—Na1—O2^iii^	83.80 (6)
C2—O2—Na1—O2^iii^	−124.92 (7)	O1—C1—Na1—O2	137.03 (7)
Na1^iii^—O2—Na1—O2^iii^	0.0	C2—C1—Na1—O2	12.47 (5)
C2—O2—Na1—O1	5.26 (6)	C2—C1—Na1—O1	−124.56 (9)

Symmetry codes: (i) −*x*, −*y*+1, −*z*+1; (ii) −*x*, *y*+1/2, −*z*+1/2; (iii) −*x*, −*y*, −*z*+1; (iv) −*x*, *y*−1/2, −*z*+1/2.

### Hydrogen-bond geometry (Å, °)

**Table:**

*D*—H···*A*	*D*—H	H···*A*	*D*···*A*	*D*—H···*A*
O2—H2···O1^iv^	0.865 (18)	1.723 (18)	2.5837 (12)	173.0 (18)
O3—H3···O1^v^	0.857 (18)	1.804 (18)	2.6575 (12)	173.7 (19)

Symmetry codes: (iv) −*x*, *y*−1/2, −*z*+1/2; (v) *x*, *y*−1, *z*.
